# Longitudinal associations between pain and cognitive decline in middle-aged and older Chinese adults: the mediating role of depressive symptoms

**DOI:** 10.3389/fpubh.2025.1526086

**Published:** 2025-03-31

**Authors:** Xia Cheng, Kun Zhang, Jiayang Liu, Jiaxin Hu, Qingxiu Yuan, Hang Cai, Hongxia Hu, Danfeng Liao, Lin Lin

**Affiliations:** ^1^School of Elderly Health, Chengdu Medical College, Chengdu, Sichuan, China; ^2^Sichuan Provincial Key Laboratory of Philosophy and Social Sciences for Intelligent Medical Care and Elderly Health Management, Chengdu, Sichuan, China; ^3^School of Nursing, Chengdu Medical College, Chengdu, Sichuan, China; ^4^The First Affiliated Hospital of Chengdu Medical College, Chengdu, Sichuan, China; ^5^Nursing Key Laboratory of Sichuan Province, Chengdu, Sichuan, China

**Keywords:** pain, cognitive dysfunction, depression, mediation analysis, cohort studies, middle-aged and older adults

## Abstract

**Objective:**

The primary aim of this scholarly investigation was to elucidate the correlation between Number of Pain Sites and cognitive decline within the older adult population. Additionally, the study sought to examine the potential mediating influence of depressive symptoms in moderating the association between pain and cognitive deterioration.

**Methods:**

We analyzed 8,835 participants aged 45 and above, with data collected from 2011 to 2018. Participants were categorized into two groups—stable and rapidly declining cognitive function—using the KML3D clustering method. Binary logistic regression analysis was conducted to examine the association between pain status, depressive symptoms, and cognitive trajectories, and mediation analysis was used to assess the mediating role of depression.

**Results:**

Multi-site pain was significantly associated with the risk of rapid cognitive decline (adjusted OR = 1.30, 95% CI: 1.14–1.48), and depressive symptoms were also a significant predictor of rapid cognitive decline (adjusted OR = 1.49, 95% CI: 1.32–1.68). Mediation analysis revealed that depression mediated the effect of pain on cognitive decline, accounting for 25.71% of the total effect.

**Conclusion:**

Our study establishes a significant longitudinal link between Number of Pain Sites and cognitive decline, mediated in part by depressive symptoms. This finding underscores the need for interventions that address pain and depression to potentially decelerate cognitive decline in older adults.

## Introduction

The older adult population worldwide is growing rapidly, with estimates suggesting it will double to 2 billion by 2050 (1). Population aging is emerging as a global phenomenon, affecting nations worldwide. While the pace and extent of demographic shifts vary among countries, the implications for public health are substantial and warrant close scrutiny (2). In China, the aging crisis is particularly severe, with the proportion of the older adult population projected to increase significantly from 12% in 2020 to 26% by 2050 (3). With the aging of the population, cognitive impairment in the older adult is becoming an increasingly significant concern (4). Xue et al. (5) conducted a systematic review and meta-analysis, revealing that 46.4% of China’s older adult population experiences subjective cognitive decline (SCD). The study identified that SCD prevalence is higher in women, older adults, individuals with lower body mass index (BMI), and those with lower levels of education (5). A separate study from rural China observed a similar trend, also noting that a significant number of individuals with SCD progress to dementia annually (6). This progression presents substantial challenges for the affected individuals and imposes considerable economic and psychological strains on caregivers and society at large (7, 8). Although cognitive deterioration is widely perceived as an irreversible condition for which a definitive therapeutic resolution is absent (9, 10), recent research shows that early diagnosis and intervention can slow cognitive decline, this can improve the quality of life for those affected and may even extend their lifespan (11, 12). Therefore, it is crucial to focus on early identification and management of modifiable risk factors (13).

Neurocognitive deficits, characterized by impairments in sensory perception, learning, memory, language, attention, and executive function, often occur in association with neurological disorders (14). Pathophysiological changes within the cerebral cortex, including both structural and functional anomalies, can lead to these deficits, providing a basis for exploring the impact of nociception on cognitive performance (15). The adverse impact of pain on cognitive performance is well-documented in an expanding academic literature (16, 17). While the impact of pain on cognitive function is evident across adulthood, this effect may be more pronounced in the older adult (18). In older adults, reduced cognitive reserve can further intensify the negative effects of pain on cognitive performance (19).

The review by Jintao Chen et al. elucidates the extensive impact of Pain on cognitive faculties in the older adult, encompassing deficits in attention, memory, executive functions, and processing velocity, which are mediated by a spectrum of biological and psychosocial pathways (20). Empirical evidence supports this association. A systematic review and meta-analysis from China has confirmed the link between pain and cognitive decline, reinforcing the growing consensus on the negative effects of pain on cognitive health (21). Furthermore, a longitudinal study among middle-aged and older adults has shown a correlation between persistent pain and memory decline, highlighting the long-term cognitive consequences of chronic pain (22). In conclusion, the aggregate data indicate a strong correlation between chronic pain and the progression of neurocognitive decline. This complex relationship may be due to shared neurobiological substrates that influence brain structure and function, emotional well-being, and lifestyle choices (20, 23).

Depressive disorders are escalating in incidence within the middle-aged and older adult demographic (24), frequently comorbid with pain and cognitive decline, thereby exacerbating cognitive impairments (25, 26). A longitudinal study, spanning 4 years and encompassing 7,968 Chinese adults over the age of 45, revealed a robust correlation between depressive disorders and the onset of cognitive impairments. The study demonstrated that enduring depressive symptoms precipitate cognitive decline, with notable gender disparities (27). Furthermore, a multitude of studies have established a significant negative correlation between depressive symptoms and cognitive function (28–30). A longitudinal analysis utilizing the Dutch Minimum Data Set for the Survey of Older People and Informal Caregivers (TOPICS-MDS) identified pain as a substantial predictor of depressive disorders, suggesting a potential mediating role of depression in the association between pain and cognitive decline (31). However, this study failed to uncover a significant link between depressive symptoms and pain, or between pain and self-reported cognitive impairment over time, indicating a possible one-way relationship between pain and depressive symptoms (31). Although the long-term dynamics between pain and cognitive function are not fully understood, they suggest a complex interplay that affects not just individual psychological health but also has significant economic implications for societal resources (32, 33).

Despite the extensive academic research that has detailed the interplay between pain, cognitive function, and depressive symptoms, our understanding of the long-term impact of pain on cognitive function among middle-aged and older adult individuals is still evolving, and the mediating role of depression in this relationship remains a subject of debate. This underscores the necessity for further research to better understand the scope of pain’s influence on cognitive decline and its broader implications for mental and cognitive health. This study leverages data from the China Health and Retirement Longitudinal Study (CHARLS) to clarify the enduring effects of pain on cognitive change in this demographic and to evaluate the potential mediating influence of depression, which bears significant implications for clinical practice.

**Figure 1:**
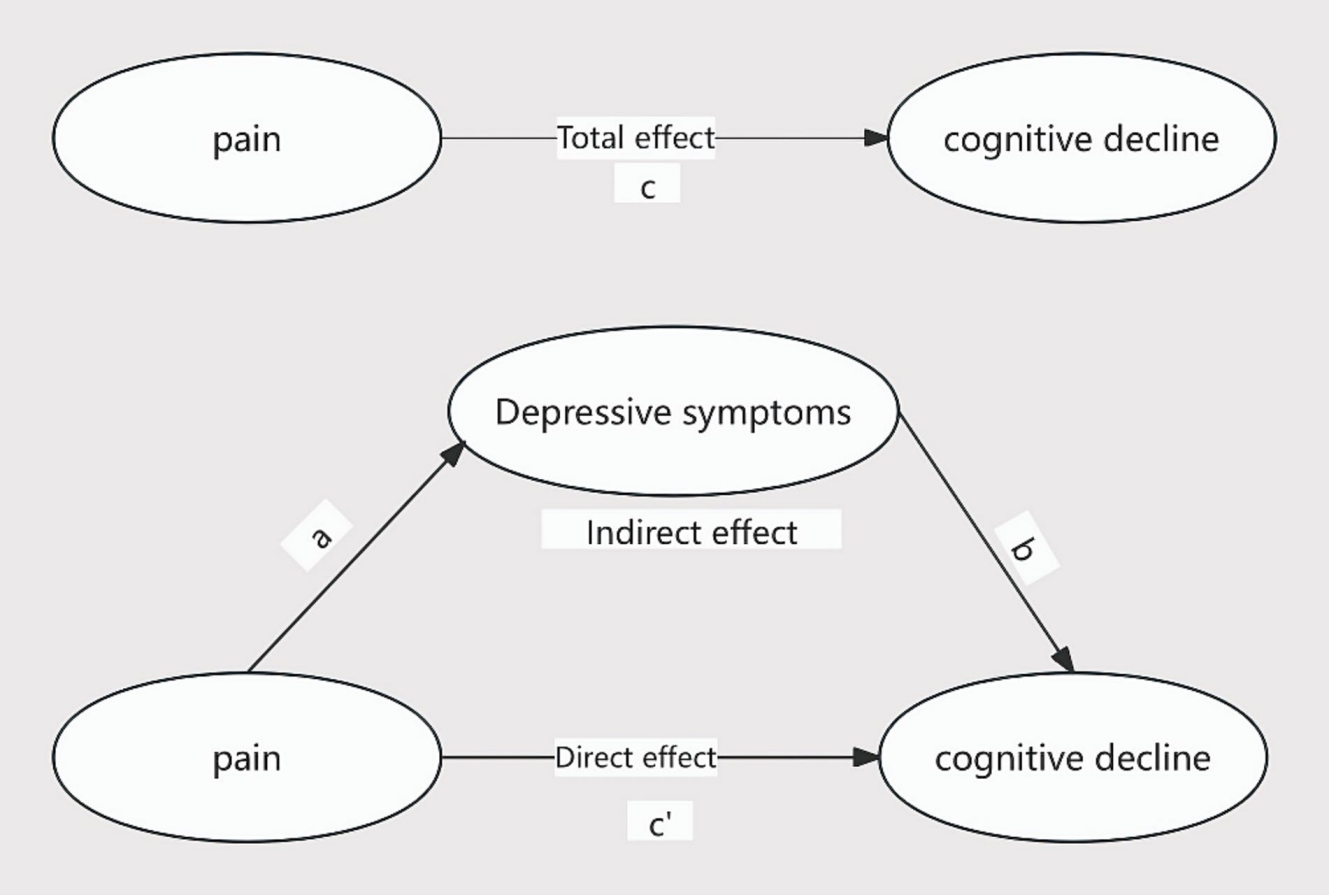
Schematic diagram of intermediary relationship.

Following a comprehensive literature review, the present investigation proposes the following three research hypotheses, and the mediation model is shown schematically in Figure 1:

*Hypothesis 1*: Within the demographic of middle-aged and older adult individuals, those afflicted with pain demonstrate a more pronounced trajectory of long-term cognitive deterioration.

*Hypothesis 2*: Within the demographic of middle-aged and older adult individuals, those with depressive disorders similarly exhibit a more pronounced trajectory of long-term cognitive deterioration.

*Hypothesis 3*: Depressive symptoms are posited to mediate, in part, the relationship between Pain and cognitive decline.

The objective of this study is to empirically validate these hypotheses, thereby furnishing theoretical underpinnings for the enhancement of health management strategies within the middle-aged and older adult demographic and establishing a scientific foundation for the formulation of clinical intervention protocols.

## Methods

### Study population

Our study utilized data from the China Health and Retirement Longitudinal Study (CHARLS), a nationally representative longitudinal cohort (34). The baseline survey was conducted between 2011 and 2012, including 17,708 adults aged 45 and older from 450 locations across China’s 28 provinces, autonomous regions, and municipalities. The survey assessed demographics, health status, cognitive function, and mental health, with follow-ups every 2 years for a total of five rounds (35). The Biomedical Ethics Committee of Peking University provided ethical approval for the study (IRB00001052-11015), and all participants provided written informed consent.

**Figure 2:**
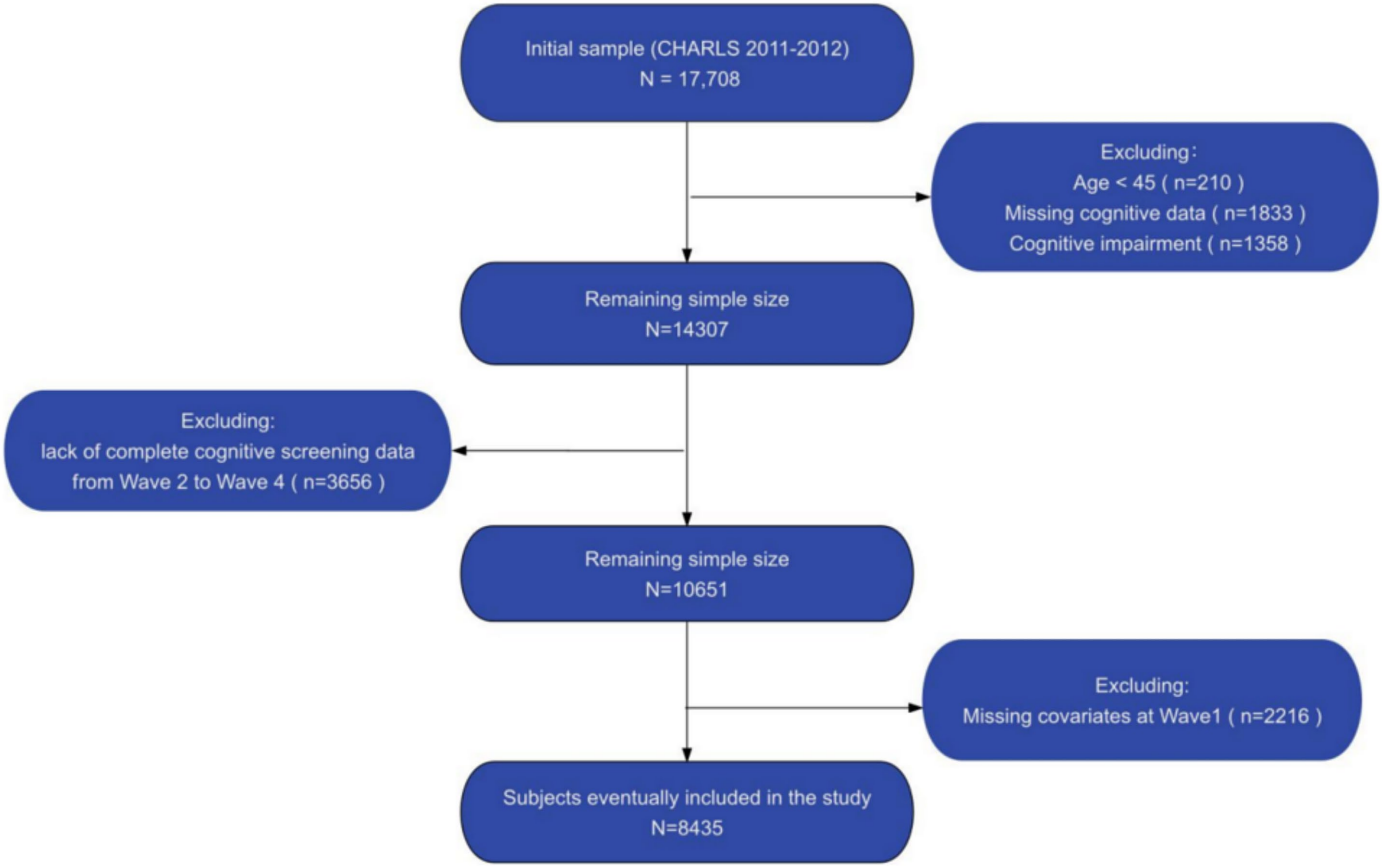
Flow chart of the selection of study participants.

In our analysis, we initially identified 8,835 participants aged 45 and older, with baseline data collected in 2011 and cognitive function assessments conducted from 2013 to 2018. Participants were excluded based on the following criteria: age below 45 years (*n* = 210), incomplete baseline cognitive assessments (*n* = 1,833), cognitive function scores at baseline more than one standard deviation below the mean (*n* = 1,358), absence of complete cognitive assessments in subsequent follow-ups (*n* = 3,656), and incomplete baseline covariate data (*n* = 2,216). The specific screening process is shown in Figure 2.

### Variable definitions

#### Assessment of cognitive function

The present study employed the Mini-Mental State Examination (MMSE) to evaluate cognitive function, a psychometric tool validated for application within Chinese demographic cohorts (36). The MMSE assesses two principal constructs: orientation and memory, as well as cognitive integrity (36). Scores for orientation and memory, ranging from 0 to 10, are determined by averaging performance across immediate and delayed recall tasks involving a list of 10 words (37). Cognitive integrity, rated on a scale from 0 to 11, is evaluated through a series of tasks that include drawing intersecting pentagons, articulating the current date, season, and day of the week, and executing a serial subtraction test (37).

This methodology enables the computation of a composite cognitive score, indicative of an individual’s overall cognitive capacity. The composite score is calculated by summing the scores for orientation and memory with those for cognitive integrity, where higher scores indicate enhanced cognitive performance (36). In this study, cognitive impairment was defined as age-associated cognitive decline (AACD) (38), characterized by scores at least one standard deviation below the age-adjusted mean (39).

#### Assessment of pain

We assessed participants’ pain experiences using two questions from the CHARLS database. First, participants were asked whether they had experienced any body pain in the past month. Those who responded affirmatively were then asked to specify the locations of their pain. We provided a list of 15 common body parts for reference, aiming to capture the distribution of pain across multiple sites (40). Subsequently, we tallied the number of pain sites participants reported, assigning one point per site, with total pain scores ranging from 0 (no pain) to 15 (pain in all sites), to gauge the severity of their pain (41). This approach allows for a more comprehensive assessment of pain distribution and severity than traditional methods, such as the 0–10 numeric rating scale (NRS), which primarily measures pain intensity without considering its spread across the body.

Building on this method of quantifying pain sites, we classified the types of pain experienced into three categories: multisite pain (pain occurring in two or more body sites simultaneously), single-site pain (pain occurring in only one site), and pain-free (no pain reported). This classification provides a clearer understanding of the complexity of pain, especially in the older adult, where pain often affects multiple areas. This method highlights the widespread nature of pain in older adults and offers a more nuanced reflection of their pain experience (41), which is essential for developing targeted pain management strategies.

#### Assessment of depression

Symptoms of depression were evaluated using the 10-item Center for Epidemiologic Studies Depression Scale (CESD-10), a scale that has established construct validity and reliability within Chinese demographic cohorts (42). The CESD-10 quantifies the presence of depressive symptoms over the preceding week across a range of ten parameters, each scored on a scale from 0 to 3, with higher scores indicating increased symptom severity (42). A CESD-10 score of 10 or above was used to identify individuals with depressive symptoms, while scores below this threshold were classified as indicating the absence of depressive symptoms (43).

#### Covariates

We controlled for various demographic and health-related covariates (44, 45), including age, gender, education level, marital status, residential area, and household consumption levels. In terms of health, we considered smoking status, alcohol consumption, Body Mass Index (BMI), insomnia severity, social participation, the number of self-reported chronic conditions, and the presence of visual or hearing impairments. Additionally, we took into account limitations in activities of daily living (ADL) and instrumental activities of daily living (IADL) (46).

#### Statistical analysis

Our study examined the longitudinal progression of cognitive function. We employed KML3D (Kernel-based Multi-Level Latent Differential) clustering to analyze the concurrent trajectories of global cognitive function, episodic memory, and cognitive integrity (47), classifying participants into two distinct groups: one with relatively stable cognitive function and another with a significant decline. KML3D is a sophisticated clustering method that leverages kernel smoothing to model complex, non-linear relationships within multidimensional data. This approach is particularly adept at managing multidimensional datasets, thereby elucidating the complex interactions among cognitive trajectories, which is essential for comprehending the intricate dynamics of cognitive health. The algorithm’s ability to handle non-linear trajectories and its robustness to outliers makes it well-suited for the analysis of longitudinal data, where individual differences and measurement errors can introduce variability (47).

We initiated our analysis using binary Logistic regression to explore the relationship between pain, depression, and cognitive decline (48), developing three sequential Logistic regression models with expanding covariate sets. The first model (Crude model) provided a baseline analysis of the unadjusted data. Model a incorporated demographic factors, including age, gender, education, residential location, marital status, and household expenditure. Model b further integrated health-related variables, encompassing smoking status, alcohol consumption, insomnia, Body Mass Index (BMI), social participation, comorbidities, and limitations in activities of daily living (ADL) and instrumental activities of daily living (IADL). Subsequently, Pearson correlation analysis was conducted to examine the linear relationships between pain, depressive symptoms, and cognitive function.

To determine how depression might mediate the link between pain and cognitive decline, we used two generalized linear models (GLM) (49). Model A looked at the relationship between depression and pain, considering certain factors. Model Y broadened this to include cognitive changes, pain, depression, and more factors. For our mediation analysis, we used R’s mediation package. We applied a nonparametric bootstrap method with 1,000 resamples to find the ACME, ADE, and TE (50). This approach gives us reliable estimates and confidence intervals without needing to assume normal distribution (51).

Furthermore, we performed stratified analyses based on distinct age, gender, educational attainment, and Body Mass Index (BMI) to determine the impact of these demographic parameters on the study outcomes (52). The statistical analyses were conducted utilizing R software, version 4.1.3. The kml3d package was instrumental in the cluster analysis of cognitive functions, leveraging the elbow method and silhouette coefficient method to determine the optimal number of clusters (53), thereby ensuring the most representative segmentation of the dataset (54).

## Results

### Participant characteristics

Table 1 presents the baseline characteristics of participants, categorized by cognitive trajectory groups. Notably, individuals of advanced age, females, those with educational attainment below the high school level, rural residents, and those with lower socioeconomic status were more likely to experience rapid cognitive decline. Additionally, participants identified as underweight, manifesting depressive symptoms, endorsing pain, exhibiting limited social interaction, experiencing vision and hearing impairments, and encountering difficulties with activities of daily living were also at an elevated risk for rapid cognitive deterioration.

**Table 1:** Characteristics of study participants stratified by cognitive status.

Variables	Trajectory of cognitive function [mean ± SD / n (%)]		*p*
	Stable Function Group (*N* = 5,189)	Rapid Decline Group (*N* = 3,246)	
Age	57.72 (7.87)	61.35 (8.42)	<0.001
Sex (female): male	2,897 (55.8)	1,181 (36.4)	<0.001
Educational level (high school or above): Less than high school	4,185 (80.7)	3,200 (98.6)	<0.001
Rural residence (non-rural): rural	3,804 (73.3)	3,006 (92.6)	<0.001
Marital status (unmarried and otherwise): married	4,855 (93.6)	2,820 (86.9)	<0.001
Social participation	2,918 (56.2)	1,486 (45.8)	<0.001
Visual impairment (no): yes	199 (3.8)	221 (6.8)	<0.001
Hearing impairment (no): yes	259 (5.0)	262 (8.1)	<0.001
Depressive symptoms (no): yes	1,438 (27.7)	1,511 (46.5)	<0.001
Current smoker (no): yes	1751 (33.7)	862 (26.6)	<0.001
Current drinker (no): yes	1,498 (28.9)	682 (21.0)	<0.001
Restriction on ADL (no): yes	237 (4.6)	323 (10.0)	<0.001
Restriction on IADL (no): yes	248 (4.8)	444 (13.7)	<0.001
consumption level			<0.001
Low	1,487 (28.7)	1,292 (39.8)	
Medium	1816 (35.0)	1,145 (35.3)	
High	1886 (36.3)	809 (24.9)	
Sleeplessness			<0.001
None	2,794 (53.8)	1,438 (44.3)	
Mild	887 (17.1)	527 (16.2)	
Moderate	689 (13.3)	550 (16.9)	
Severe	819 (15.8)	731 (22.5)	
BMI (kg/m2)			<0.001
Underweight	158 (3.0)	258 (7.9)	
Normal	2,983 (57.5)	1923 (59.2)	
Overweight	1,518 (29.3)	789 (24.3)	
Obesity	530 (10.2)	276 (8.5)	
Comorbidity			<0.001
0	1820 (35.1)	1,003 (30.9)	
1	1,557 (30.0)	969 (29.9)	
≥ 2	1812 (34.9)	1,274 (39.2)	
Physical pain			<0.001
No pain	3,800 (73.2)	1940 (59.8)	
Single site pain	362 (7.0)	238 (7.3)	
Multisite pain	1,027 (19.8)	1,068 (32.9)	

BMI, Body mass index; ADL, Activities of Daily Living; IADL, Instrumental Activities of Daily Living: X, Arithmetic mean; SD, Standard deviation; *p* value was based on *χ*^2^ or analysis of variance or Mann–Whitney. ‘Age’ is shown as mean ± SD, all other variables are shown as *n* (%).

### Cognitive change trajectories

**Figure 3:**
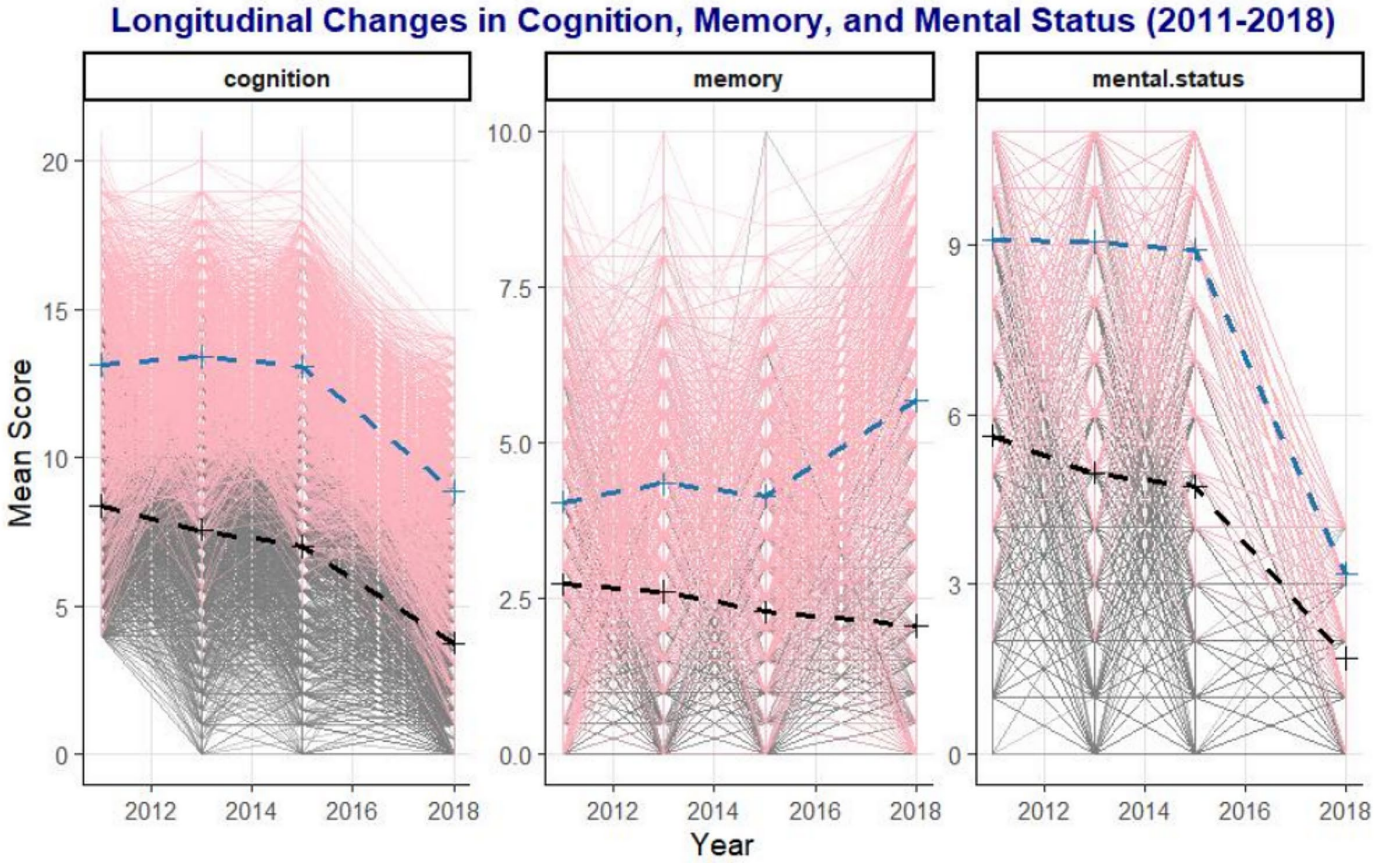
Longitudinal change trajectories based on cognition, memory, and mental state.

Utilizing the KML3D clustering approach, our study delineated the longitudinal trajectories of episodic memory, cognitive integrity, and composite cognitive scores among participants. This analysis revealed two distinct participant groups with divergent cognitive trends (as illustrated in Figure 3). One group demonstrated preservation of cognitive performance (represented by the blue line), while the other exhibited significant cognitive decline (represented by the black line). The background line graphs in Figure 3 present the individual trajectories for cognitive performance, episodic memory, and cognitive integrity, highlighting the variability in cognitive trajectories over time (47). The distinct patterns identified by KML3D clustering underscore the heterogeneity in cognitive decline rates and underscore the method’s utility in identifying individuals at risk of accelerated cognitive deterioration. These line graphs effectively capture the subtleties of cognitive change, aligning with our methodological intent to employ KML3D for a more nuanced understanding of cognitive dynamics.

**Figure 4:**
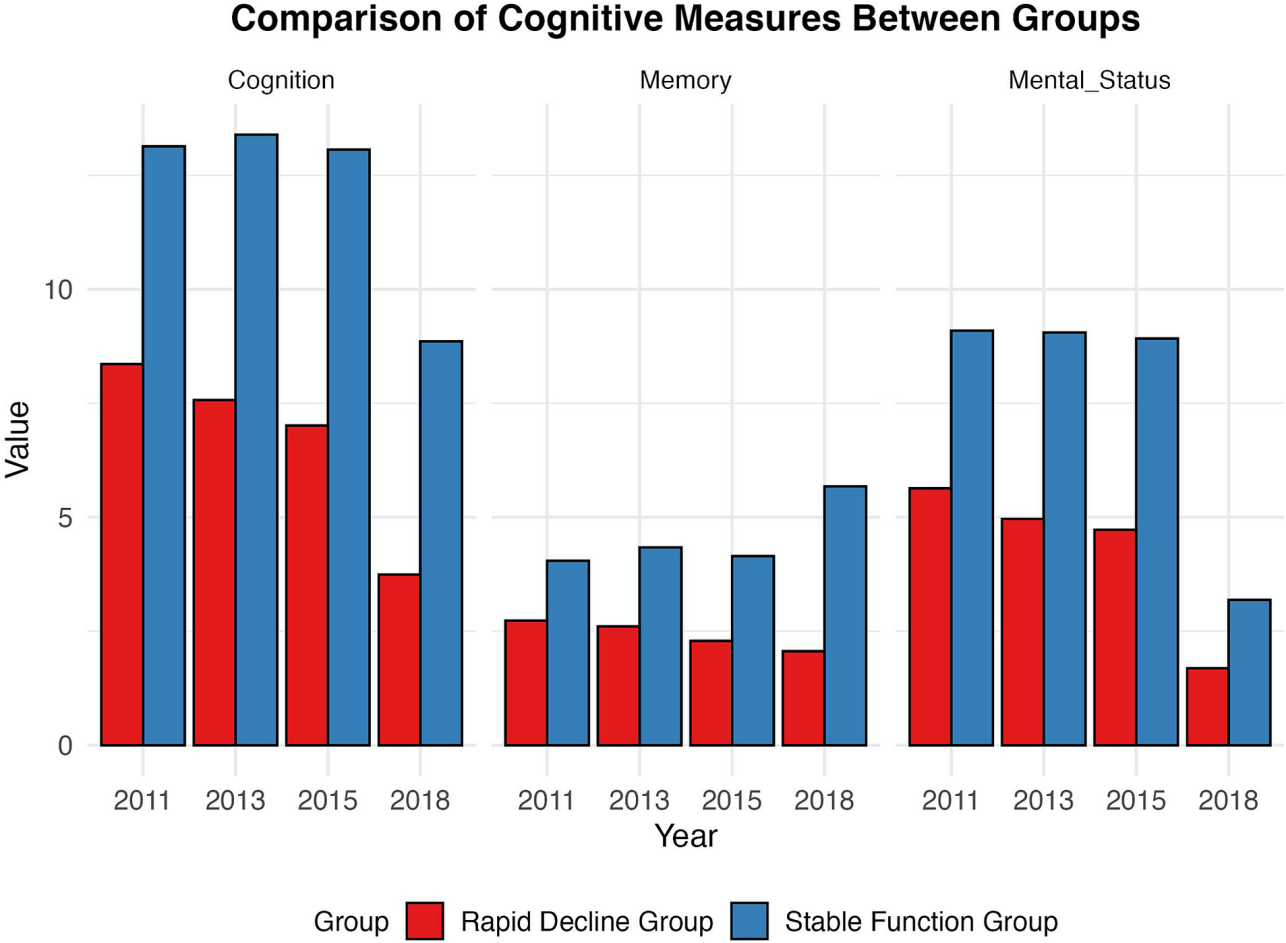
Between-group comparisons of cognitive function.

Figure 4 displays the mean scores for global cognitive function, episodic memory, and affective state across two cohorts over four data collection waves, revealing significant disparities. The cohort with stable cognitive function, despite a decline in affective state and a slight decrease in cognitive scores, showed superior performance compared to the cohort with rapid cognitive decline, particularly in episodic memory, which is crucial for maintaining their cognitive stability (55). Conversely, the cohort experiencing rapid cognitive decline demonstrated a consistent deterioration in both memory and affective state, indicating a potential lack of effective cognitive preservation strategies and a negative trend in cognitive performance within this demographic (56).

### Associations of pain, depression, and cognitive trajectories

This study aimed to ascertain the effects of pain status and depressive symptoms on the risk of rapid cognitive decline. Through binary logistic regression analysis, we assessed the relationship between these variables and the probability of cognitive deterioration. The results (Table 2) indicated that, after controlling for basic socio-demographic variables (model a), participants with multi-site pain had a significantly increased risk of rapid cognitive decline (OR = 1.66, 95% CI: 1.48–1.86, *p* < 0.001). This risk remained significant even after adjusting for additional lifestyle and health status variables (model b), with an odds ratio of 1.30 (95% CI: 1.14–1.48, *p* < 0.001). In contrast, single-site pain was not significantly associated with an increased risk of rapid cognitive decline after adjusting for covariates (model b), with an odds ratio of 1.03 (95% CI: 0.84–1.25, *p* = 0.795).

**Table 2:** Binary logistic regression results for pain, depression and cognitive trajectories.

Variable	Crude Model		Model^a^		Model^b^	
	OR (95% CI)	*p*	OR (95% CI)	*p*	OR (95% CI)	*p*
Pain status						
No pain	1.00 (1.00,1.00)	Ref.	1.00 (1.00,1.00)	Ref.	1.00 (1.00,1.00)	Ref.
Single site pain	1.29 (1.08–1.53)	0.004	1.20 (0.99–1.45)	0.059	1.03 (0.84–1.25)	0.795
Multisite pain	2.04 (1.84–2.56)	<0.001	1.66 (1.48–1.86)	<0.001	1.30 (1.14–1.48)	<0.001
Depression	2.27 (2.07–2.49)	<0.001	1.83 (1.65–2.02)	<0.001	1.49 (1.32–1.68)	<0.001
CESD (Per SD)	1.08 (1.07–1.09)	<0.001	1.06 (1.05–1.07)	<0.001	1.05 (1.03–1.06)	<0.001

OR, Odds Ratio; CI, Confidence Interval; Ref., Reference category; CESD, Center for Epidemiologic Studies Depression Scale; Per SD, Per Standard Deviation. Crude model no adjustments were made.

a Adjusted for Age, Gender, Educational level, Residence, Marital status, consumption level.

b Further adjusted for Smoking status, Drinking status, Sleeplessness, BMI, Social participation, Comorbidity, Visual impairment, Hearing impairment, Restriction on ADL, Restriction on IADL.

Moreover, depressive symptoms were identified as a significant predictor of rapid cognitive decline (model b: OR = 1.49, 95% CI: 1.32–1.68, *p* < 0.001). Notably, each one standard deviation increase in the CESD-10 scale score was associated with a 5% increase in the risk of rapid cognitive decline (OR = 1.05, 95% CI: 1.03–1.06, *p* < 0.001), suggesting that even minor increases in depressive symptoms could contribute to a heightened risk of cognitive decline. These findings highlight the necessity for stringent management of pain and depressive symptoms to reduce the risk of rapid cognitive decline.

Pearson correlation analysis, as presented in Table 3, revealed a significant negative correlation between pain and cognitive function (*r* = −0.158, *p* < 0.01), indicating that higher pain levels were associated with poorer cognitive function. Additionally, a significant positive correlation was found between pain and depressive symptoms (*r* = 0.410, *p* < 0.01), suggesting that higher pain levels were associated with more severe depressive symptoms.

**Table 3:** Pearson’s bivariate correlation matrix for pain, depressive symptoms, and cognitive function.

Variable	Cognitive function	Depressive symptoms	Pain scores
Cognitive function	1		
Depressive symptoms	−0.282^***^	1	
Pain scores	−0.158^***^	0.410^***^	1

^***^p < 0.01.

### Depression as a mediator in the association between pain and cognitive decline

The mediation analysis revealed a substantial indirect effect of pain on cognitive deterioration, mediated by depression. The Total Effect (TE) was estimated at 0.0639 (95% confidence interval [CI]: 0.0437–0.0800), with a *p*-value <0.001, indicating a significant association between pain and cognitive decline. When controlling for depression, the Average Direct Effect (ADE) of pain on cognitive decline was 0.0475 (95% CI: 0.0277–0.0600), also with a *p*-value <0.001, which suggests that pain retains a detrimental direct effect on cognitive decline.

Furthermore, the Average Causal Mediation Effect (ACME) attributed to depression was 0.0164 (95% CI: 0.0119–0.0200), with a *p*-value <0.001, confirming the mediating role of depression in the pain-cognitive decline relationship. Depression mediated 25.71% (95% CI: 16.98–37.00%) of the total effect, with a p-value <0.001, highlighting the significant role of depression in this context. A comprehensive overview of these results is presented in Table 4.

**Table 4:** Results of the mediation analysis of pain through depression on cognitive decline.

Effect	Estimate (95%CI)	*p*
Total Effect (TE)	0.0639 (0.0437, 0.0800)	<0.001
Average Direct Effect (ADE)	0.0475 (0.0277, 0.0600)	<0.001
Average Causal Mediation Effect (ACME)	0.0164 (0.0119, 0.0200)	<0.001
Percentage Mediated (PM)	0.2571 (0.1698, 0.3700)	<0.001

TE, Total Effect; ADE, Average Direct Effect; ACME, Average Causal Mediation Effect; PM, Percentage Mediated; All *p*-values are less than 0.001, indicating statistical significance.

### Findings from stratified analyses

Our stratified analyses showed that the relationship between pain and the risk of cognitive decline varies among different demographic groups. For those aged 45–59 and 70–79, both single-site pain [odds ratio (OR) 1.50, 95% confidence interval (CI) 1.17–1.92; OR 1.69, 95% CI 1.03–2.80] and multi-site pain (OR 1.98, 95% CI 1.70–2.30; OR 2.42, 95% CI 1.81–3.26) were associated with a higher risk of cognitive decline. However, in the 60–69 and 80+ age groups, only multi-site pain was significantly linked to an increased risk (OR 2.02, 95% CI 1.71–2.38; OR 2.81, 95% CI 0.97–10.23, with the confidence interval including 1 for the 80+ age group, which may indicate a lack of statistical significance).

**Figure 5:**
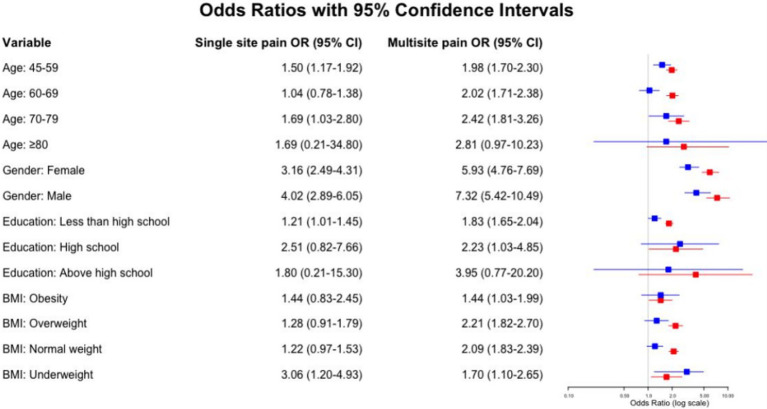
Stratified analysis of the relationship between pain and rapid cognitive decline.

Gender analysis revealed that multi-site pain significantly increased the risk of cognitive decline in females (OR 5.93, 95% CI 4.76–7.69) and males (OR 7.32, 95% CI 5.42–10.49). In individuals with a high school education or less, single-site pain (OR 1.21, 95% CI 1.01–1.45) and multi-site pain (OR 1.83, 95% CI 1.65–2.04) were both associated with a higher risk of cognitive decline. In terms of BMI, multi-site pain was significantly associated with cognitive decline risk in overweight individuals (OR 2.21, 95% CI 1.82–2.70) and in those with normal weight (OR 2.09, 95% CI 1.83–2.39). Figure 5 provides a detailed analysis and a visual representation of these results.

## Discussion

This investigation employed longitudinal data from the China Health and Retirement Longitudinal Study (CHARLS) (35) and applied the K-means clustering algorithm to delineate two discrete trajectories of cognitive function. The first trajectory was characterized by the maintenance of cognitive performance, whereas the second was marked by a notable deterioration. These findings corroborate the extant scholarly discourse on cognitive heterogeneity (57), reinforcing the conceptualization of cognitive decline as a multifactorial phenomenon that encompasses diverse determinants and dimensions (58).

The findings of our study demonstrate a robust association between polymodal pain and the risk of cognitive decline, which remains significant after controlling for various potential confounders. This result supports the hypothesis that pain adversely affects cognitive function, aligning with previous research (17, 20, 59). The extant literature suggests that pain is associated with alterations in brain structure and function, particularly within regions critical for executive functions and memory, such as the prefrontal cortex and hippocampus (60, 61). Furthermore, pain may exacerbate cognitive decline by impacting sleep quality, lifestyle, and emotional well-being (62). The Pearson correlation analysis confirmed significant correlations among pain sites, depressive symptoms, and cognitive function, reinforcing the complex interactions observed in our logistic regression analyses. Similar findings were reported in a study using the Study of Health, Aging, and Retirement in Europe (SHARE) dataset, which employed multilevel linear regression models (63). Another cohort study involving the China Health and Retirement Longitudinal Study (CHARLS) and the English Longitudinal Study of Aging (ELSA) also confirmed a significant bidirectional association between pain and depression (64). These studies suggest a complex interplay among pain, depression, and cognitive function.

Depressive symptoms are strong predictors of rapid cognitive decline, as indicated by the study (65). Our results align with the current literature, emphasizing the impact of affective states on cognitive health. Table 2 demonstrates that for every standard deviation increase measured by the CESD-10 scale, the risk of rapid cognitive decline increases by 5%. This suggests that even mild depressive symptoms can negatively affect cognitive performance. This finding corroborates the established dose–response relationship between the severity of depressive symptoms and cognitive decline, as observed in previous studies (66).

Mediation analyses, as presented in Table 4, demonstrated significant partial mediation by depression in the relationship between pain and cognitive decline. Depression was found to account for 25.71% of the total effect, highlighting the substantial impact of depressive symptoms on cognitive health, suggesting that interventions targeting depression could potentially mitigate the risk of cognitive decline linked to pain. This finding corroborates the significant influence of emotional states on cognitive health, as confirmed by previous studies (67), and provides novel insights for refining pain management strategies.

Our study has several strengths. Firstly, we employed longitudinal data analysis to explore the intricate interplay between pain, depressive symptoms, and cognitive decline. This methodology allows for a comprehensive assessment of the long-term effects of pain and depressive symptoms on cognitive function. Secondly, our study extends beyond examining simple bivariate relationships, further substantiating the mediating role of depressive disorders in the relationship between pain and cognitive senescence. This advancement provides a deeper insight into the mechanisms linking pain to cognitive decline. Additionally, our research provides a theoretical framework for the development of personalized preventive strategies. By conducting stratified analyses that account for variables such as age, gender, educational attainment, and body mass index (BMI), we have identified key demographic and physiological factors that can inform tailored preventive measures. These findings underscore the value of our study in contributing to the development of targeted interventions aimed at mitigating the risk of cognitive decline.

This study has several limitations. Firstly, we utilized the MMSE assessment scale rather than more objective neuroimaging techniques or biomarkers (such as blood-based biomarkers), which may limit the accuracy and generalizability of our findings. Future research should adopt the gold standard for diagnosing cognitive impairment by incorporating a combination of clinical assessments, neuropsychological testing, laboratory analyses, and imaging techniques. This approach would allow for more precise cognitive measurements and enable a more comprehensive exploration of the factors influencing cognitive decline. Secondly, this study focused solely on the impact of pain distribution on cognitive decline, without considering the duration of pain or the differential effects of acute versus chronic pain. Future studies should incorporate a broader range of pain characteristics to provide a more detailed and comprehensive analysis. Additionally, the data in this study were sourced exclusively from the CHARLS database. Given the unique characteristics of the middle-aged and older adult population in China, our findings may not be directly applicable to other ethnic or geographical groups. Therefore, caution should be exercised when extrapolating our results. Future research could validate these findings by conducting multi-database association analyses across different populations, ensuring broader applicability and generalizability of the results. Finally, the assessment of pain and depression in this study was limited to baseline measurements, with no follow-up evaluations. This limitation restricts our understanding of the dynamic relationship between pain, depression, and cognitive function. Future research should incorporate long-term follow-up studies or intervention trials to more deeply investigate causal relationships. Furthermore, evaluating the efficacy of integrated intervention strategies, such as pain management and psychological therapies, in mitigating cognitive risks in middle-aged and older adult populations would provide valuable insights. These efforts are crucial for advancing our understanding of healthy aging and for developing effective strategies to promote cognitive health.

## Conclusion

This scholarly investigation elucidates the pivotal role of pain in cognitive senescence among middle-aged and geriatric individuals and underscores the mediating function of depressive disorders. The research introduces novel insights into the complex interplay among pain, depressive disorders, and cognitive decay. Despite inherent limitations, the findings have theoretical relevance and potential clinical implications, particularly for concurrent therapeutic interventions for pain and depressive disorders. Future research should focus on delineating the specific mechanisms by which pain affects cognitive function and assess the feasibility of personalized therapeutics through clinical intervention studies. Such inquiries are essential for enhancing the efficacy of preventive strategies against cognitive decline and optimizing health management protocols for the older adult.

## Data Availability

Publicly available datasets were analyzed in this study. This data can be found here: https://charls.charlsdata.com/pages/data/111/zh-cn.html.
